# Tracking conflict and emotions with a computational qualitative discourse analytic support approach

**DOI:** 10.1371/journal.pone.0251186

**Published:** 2021-05-13

**Authors:** Nikodem Rybak, Daniel J. Angus

**Affiliations:** 1 School of Information Technology and Electrical Engineering, University of Queensland, St Lucia, Australia; 2 Digital Media Research Centre, Queensland University of Technology, Brisbane, Australia; National Institutes of Health, UNITED STATES

## Abstract

Accurate inferences of the emotional state of conversation participants can be critical in shaping analysis and interpretation of conversational exchanges. In qualitative analyses of discourse, most labelling of the perceived emotional state of conversation participants is performed by hand, and is limited to selected moments where an analyst may believe that emotional information is valuable for interpretation. This reliance on manual labelling processes can have implications for repeatability and objectivity, both in terms of accuracy, but also in terms of changes in emotional state that might go unnoticed. In this paper we introduce a qualitative discourse analytic support method intended to support the labelling of emotional state of conversational participants over time. We demonstrate the utility of the technique using a suite of well-studied broadcast interviews, taking a particular focus on identifying instances of inter-speaker conflict. Our findings indicate that this two-step machine learning approach can help decode how moments of conflict arise, sustain, and are resolved through the mapping of emotion over time. We show how such a method can provide useful evidence of the change in emotional state by interlocutors which could be useful to prompt and support further in-depth study.

## Introduction

Human communication is a dynamic, complex and reciprocal process that relies on interlocutors encoding and decoding communicative acts via multiple modalities. Advances in computational processing in the previous decades have motivated interest in the development of computational techniques and tools for aiding the analysis of conversational exchanges, many belonging to a family of techniques called Computer Assisted Qualitative Data AnalysiS (CAQDAS) [1–3]. Most CAQDAS techniques do not seek to replace the need for human judgement or interpretation, rather instead to augment human judgements [4].

In this paper, we propose a computational affect detection technique, aimed at assisting the analysis of human emotion in communication. Emotions play essential roles in the development, preservation, and evolvement of important social constructs, an example being conflict, which arise from human-to-human interactions on various time scales. The proposed technique draws on insights from communication science, psychology and sociology to understand the role of non-verbal communication modalities.

The proposed technique uses current state-of-the-art deep neural networks, combined with statistical methods for automatic classification of the affective state of interlocutors, based on complex non-verbal interaction dynamics. The system extracts seven fundamental emotions from facial expressions of participants and then uses a customized linear regression classifier to find patterns in which these emotions correlate to conflict. This novel technique enables the prediction of conflict levels within a face-to-face interaction over the entire time course of the interaction. Importantly the technique is designed to be used with low quality broadcast video, such as what someone might obtain from an online video service such as YouTube, and which is commonly used by many discourse analysis scholars. This is an important distinction given how most alternative emotion recognition systems rely on high quality continuous full-frontal face and/or audio recording, making them unsuitable for post-hoc analysis of broadcast interview data.

The paper begins with an overview of conflict in broadcast interviewing, theories of emotion and facial expressiveness, and machine learning. We then outline the emotion classification system in detail, and introduce the data sets used for the training of the machine learning classification system. We demonstrate the system on a series of well-studied broadcast interview videos to both determine the ability of the system to correctly classify points of interest (conflict), and to showcase potential workflows and use in future broadcast discourse studies. We then offer concluding thoughts regarding the use of this technology in future studies and implications for discourse analysis practice. Our goals for this study are two-fold, firstly to propose a useful technology for supporting discourse analysis, and secondly to offer an introduction to deep neural network technologies for a social scientific audience.

### Broadcast–Conflict context

Interviews are a salient genre across a range of broadcast and print media output and there has been frequent use of interview in confessional and entertainment formats [5]. One major characteristic of media broadcast interviews as a generic form is brought out in the way they position talk for the benefit of an overhearing audience. It is common knowledge that both the interviewer and interviewee expect that what they say will be appraised by their interlocutor but also the many others who can access the broadcast. Broadcast interviews are characterized with clear differentiation or pure pre-allocation of roles whereby one speaker asks questions while the other answers them. On most occasions, the speaker who asks questions is determined from an institutional point of view and is tasked with setting the agenda [6].

Broadcast interviews have important implications for the public’s understanding of news and current affairs, particularly because they are a manifestation of a widely used public genre that offers journalism an important channel for dissemination of quotable material to underpin the news. Broadcast interviews have been previously grouped into four interview types: ordinary people who are caught up in the news, interviews with correspondents who report and comment, interviews with experts who explain and inform, and interviews with principals (public figures) often for the purpose of accountability [5].

How conflict arises, or how it is managed by participants within a broadcast interview context has been the subject of several studies. [7] provides an authoritative account of conflict in talkback radio interviews, with findings that generalize across most broadcast interview contexts. [7] reveals how conflict is an interactional phenomenon, and that particularly in a broadcast setting such interactions have asymmetrical power dynamics that can frame the manner in which conflict is initiated, amplified, moderated, and eventually concluded. Revealing the wider context and timelines of the emergence of conflict, how it is enacted, and eventually how it concludes is therefore an important methodological consideration for the study of broadcast discourse.

### Facial expressiveness

Disciplines such as social psychology [8], cognitive science [9], systems engineering [10], and communication [11] each examine the behavioural patterns of groups in social and professional settings by describing various communication stages, behaviours, and of specific interest here: modalities of communication.

Although the speech/verbal modality is considered a very robust communication channel in any social interaction, it is well established that when available, nonverbal modalities are important in almost any spoken interaction [12,13]. Nonverbal modalities involve observable changes such as hand and body gestures, eye movements, and perhaps most significantly, facial expressions [14].

Previous research on the dynamics of nonverbal interaction indicates that while many social cues are intentional (i.e., a conscious drive to accomplish specific aim within the discussion), many of the other signals are the consequence of unintentional and unconscious processes [15]. Additionally, humans involved in an act of communication can recognize social cues quickly, unconsciously and with high accuracy. There is also a significant work related to more complex processing of these signals in social situations related to social identities, high level relationships of our social world based on deeper internal conditions of participants [16]. It is known that social behaviour and social constructs are regulated by the demonstration and recognition of nonverbal signals, in many circumstances without depending on spoken language at all [15].

### Machine learning/deep learning

The main objective of machine learning is to deliver mathematical models and algorithmic solutions that can be utilized to solve problems that humans accomplish unthinkingly and most often unconsciously, but are too demanding to be represented as standard mathematical problems. A class of such problems is recognizing the patterns of facial expressions within an image containing a human face. This function can be performed by a mature human brain with almost no effort, but remains a daunting task to be represented as a mathematical problem due to its huge and mostly unconstrained complexity.

Artificial Neural Networks (ANNs) are a specific class of ML procedures that specialize in pattern recognition, conceptually based on the manner in which biological neural structures process signals. ANNs are sets of mathematical tools recently utilized with great success to tackle various classification problems, including speech, images, medical diagnostics, and other signal data.

Deep learning (deep neural networks—DNNs) are the latest development in ANNs. Deep learning methods and techniques scale up the size and complexity of previous ANNs to create increasingly richer functionality.

A typical instance of applied deep learning is the Convolutional Neural Network (CNN) [17] applied to computer vison tasks like handwritten character recognition which automatically learns discriminating patterns from images by sequentially assembling layers vertically. Lower levels Layers of the trained network represent configurations of corners and edges, whereas high level layers learn more abstract relations between shapes to discriminate between characters. In many applications, CNNs are now considered the most powerful image classifier and are currently responsible for pushing the state-of-the-art forward in computer vision subfields that leverage machine learning [18].

To understand how deep learning has revolutionized the field of computer vision requires briefly looking into previous solutions. In the past, computer vision used mainly hand-engineered features to quantify the contents of an image. For example, previous algorithms executed feature extraction separately on each image in a dataset according to predefined image descriptors, returning a vector to quantify the content of an image. The next step in the process was to extract significant structures from a dataset, by performing “flat” statistical inference on received vectors.

Traditional hand-engineered image descriptors endeavoured to encode features such as colour, texture or shape [19]. Other broadly utilized methods were key point detectors (FAST, Harris, DoG to name a few) and local invariant descriptors (e.g. SIFT, SURF, BRIEF, ORB, etc.) [20]. Some methods like Histogram of Oriented Gradients (HOG) proved to be efficient at detecting objects in images when the rotation of an object within an image did not significantly vary from what the classifier was trained on [21].

In all abovementioned approaches, a process was predefined to encode the exact characteristics of an image (i.e., colour, shape, texture etc.) and quantify it. Given an input matrix of pixels, one would apply this hand-defined algorithm to the pixels, and in return receive a feature vector quantifying the image contents—the image pixels themselves did not serve a purpose other than being inputs to the feature extraction process. The feature vectors that resulted from feature extraction were what was truly important as they served as inputs to machine learning models.

The Convolutional Neural Network approach used in our method uses a different approach for locating patterns within an image. In place of hand-engineered instructions to extract specific features, these features are repeatedly learned from the training process completed on thousands of examples for each instance. More importantly, this deep learning approach is based on multi-level representation learning, achieved by combining relatively simple nonlinear segments that each transform the representation at one step (starting with the raw input of pixels) into a representation at a higher, more abstract level. The key aspect of deep learning is that patterns extracted on a level of each layer are not designed by human engineers. They are automatically learned from data itself utilizing a general-purpose learning procedure.

While biologically inspired, deep neural networks are not realistic models of brain structures. However, they mimic the functions of the brain in regard to generalization and specialization of patterns that can be used to group or differentiate images, sounds or other inputs, and have proved to be useful in examining an accuracy of the brain behaviour models [22].

In summary, the aim of machine learning is to build computers that can learn from experience, and determine internal rules for how objects or phenomena can be grouped according to their latent features. Using deep learning is to define a recognition problem as a hierarchy of representations. Representations in the lower layers of the model encode rudimentary characteristics, while higher level layers use these elementary features to generate more abstract concepts. This hierarchical learning allows us to remove the hand-designed feature extraction process and treat CNNs as end-to-end learners which frequently results in an efficient classifier.

### Digitizing emotions

Communicating, recognizing and understanding emotions can have significant impact on the pragmatics of social interaction. Human-machine interaction (HMI) researchers have long investigated the potential of designing algorithms to model affects and augment verbal and non-verbal exchanges [23]. However, computer-based affect classification remains a complex issue due to the mathematical complexity of human emotional representations [24]. Therefore, the search to invent novel methods to establish more precisely the emotions of a subject has become a major interest in the field of machine learning and its subfield, deep learning [25,26].

Over last twenty years, disciplines including political science [27], sociology [28] as well as social psychology [29] have moved their attention to integrative approaches, which merge aspects of purely cognitive study with research on emotions. This shift is in part an outcome of experimental evidence that emotions are a fundamental component of social interactions and that the understanding of their patterns is a precondition for the research of individual and collective behaviours. There are, however, very few well-established datasets on how various individuals express their affective states via facial expressions in the specific circumstance of conflict with an interlocutor. Thus, to correctly define and classify the manifestations of conflict, we examined an approach in which these facial expressions are described using a combination of rudimentary affective states as determined by Ekman and Heider [30].

Ekman and Heider’s framework presents seven primary emotional states: anger, disgust, contempt, fear, happiness, sadness and surprise [30]; originally presented as six states, contempt was added years later [31]. They are theoretical concepts–perfect states; the attributes and qualities of which can be defined innately. These primary affects are intrinsic phenomena that occur corollary of an external stimulus, and are typically sustained for a brief interval. In contrast, other affective states and more complex emotion-based behaviour are considered derivative states under this framework. They arise as compounds or coalescences of rudimentary affects. As stated by Ekman and Heider, those emotions are universal and can be observed amongst all cultures, although we note that this is a contested claim.

As suggested by Mehrabian’s comparative examinations, the most informative modalities for the non-contact examination of psychological processes are facial expressions [32]. According to this work on affect non-verbal manifestations, facial expression conveys up to 55% of information on subject’s emotional state. Hence in this study we combine Ekman and Heider’s emotion model with facial expression data to create our baseline data set.

It is important to note that this model of emotions could be readily replaced with an alternative model, and likewise so could the modality of emotional expression. Such replacements are contingent on the availability of labelled examples for each discrete (or indeed composite) emotional states or otherwise to train the classifier. It is also important to note that the training data selected has an important role to play in the general applicability of the resulting trained model, and this is a moment where racial, gender and other bias can find its way into such a system if not explicitly controlled and addressed.

## Materials and methods

### System overview

We employed an empirical framework to determine the suitability of our deep learning emotion and conflict recognition approach, and the qualitative support framework. Our DCNN model has been designed, trained and verified on gold-standard datasets to evaluate its recognition accuracy and overall performance, and case studies of broadcast interviews used to deliver insights into its suitability for assisting in the task of analysis of broadcast discourse.

**Fig 1:**
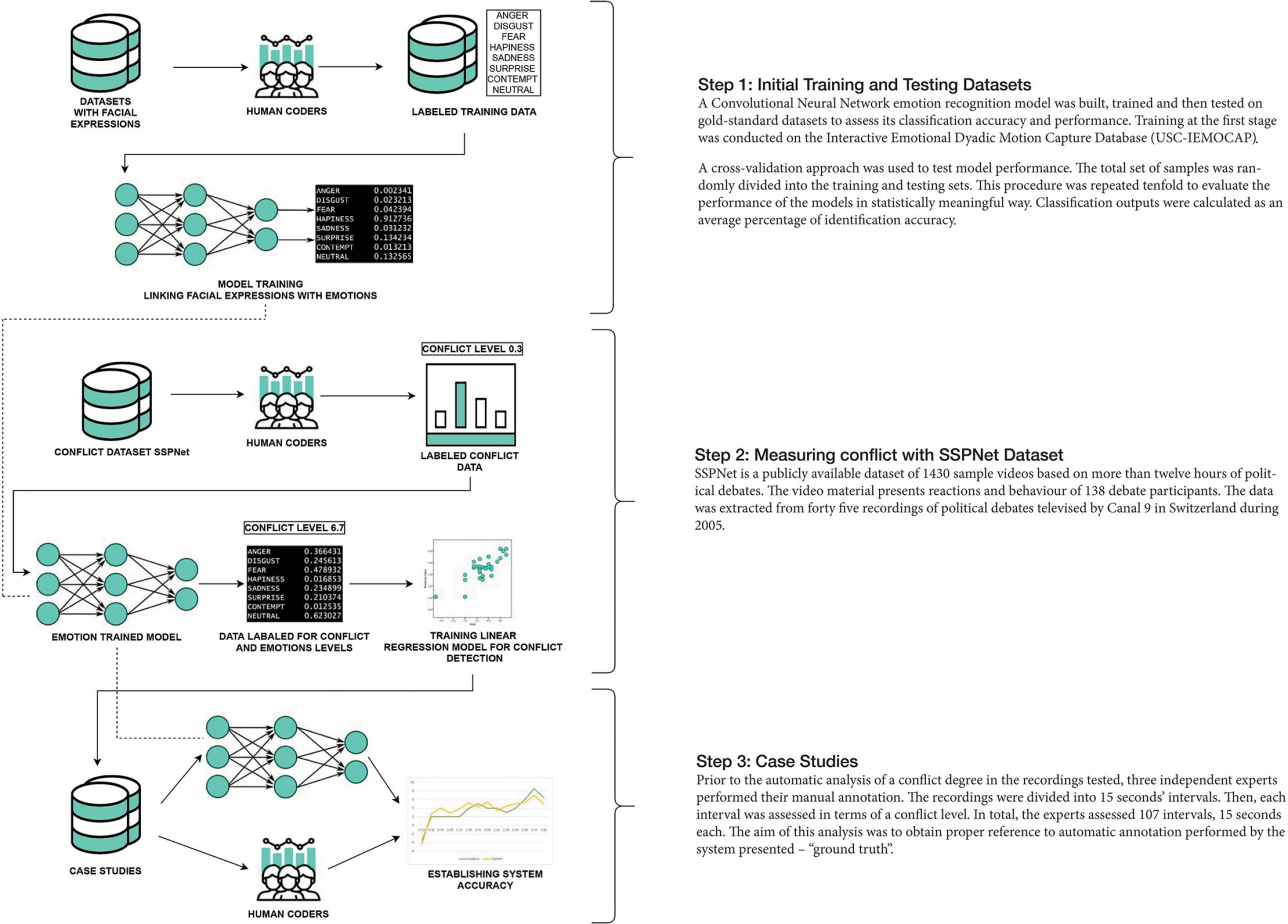
System overview. A summary of our hierarchical approach for automatic conflict measurement. Training of this conflict prediction model was completed using the SSPNet Conflict Corpus. Each input video frame is classified across the 7 dimensions of emotion. The vectors of emotions are then used as a basis for prediction of the level of conflict.

The use of data in our approach is somewhat different from other discourse analysis studies, as much of the data we are using is for the purpose of training the computational system, and testing the accuracy of its classifications, while some is reserved for more in-depth qualitative analysis. These datasets are explained in their relevant sections which detail the different aspects of our proposed system (see Fig 1 for a system-wide overview).

### Emotion classification training datasets

The Interactive Emotional Dyadic Motion Capture Database (USC-IEMOCAP) provides the first tranche of data for training our computational models. The USC-IEMOCAP is “an acted, multimodal and multi-speaker database and contains approximately 12 hours of audio-visual data, including video, speech, motion capture of face, text transcriptions. 10 professional actors (5 males, 5 females) perform improvisations or scripted scenarios, specifically selected to elicit emotional expressions” [33].

The sessions are divided into segments of utterances. Each section is labelled by three human experts, with agreement reached in an average of 74.6% of cases. We used only the annotations of the samples that received a majority-consensus, removing segments with low inter-coder reliability. The classes of happiness and excitement have been combined into one category to offset the problem of uneven number of samples within categories. As a result, every class consists of approximately same quantity of 1,673 segments.

The USC-IEMOCAP dataset has been utilised as a principal source for the training process owing to its substantial size. However, in the sample footages, the subjects are located at a distance with faces not always fully observable. Therefore, to enhance the process of the frontal face pose we supplemented the training process with samples from FACES [34] and RaFD [35] databases. The FACES dataset entails 179 subjects whereas the RaFD carries examples from 49 subjects. The images in both databases are in an upright and fully frontal pose. Similar to above we have only used samples from this dataset that are of a high inter-coder reliability.

### Face detection and tracking

Before the system can attempt to classify facial emotion information, it must be able to locate a face within a video frame. To detect faces within a given image sample, the implementation of the CascadeClassifier class object from the OpenCV library was used [36].

The following are the stages of the face extraction process:

1. Searching for the face occurrence area in the analyzed video frame.
2. Eye pair searches in all parts of the image found, with only those areas where exactly one pair of eyes are detected for further processing.
3. Calculation of the frame dimensions and position based on the size of the rectangle surrounding the pair of eyes found and the class calling parameters for framing.
4. Cutting the calculated frame from the input image and scaling it to specific dimensions.

According to [37], the HaarScale parameter corresponds to the scaling factor of the classifiers in subsequent algorithm iterations. The larger it is, the greater the difference between the size of classifiers in individual steps. This allows speeding up calculations, however, increasing the risk of omitting a face. The HaarScale parameter is passed to the cvHaarDetectObjects function as an argument named scaleFactor. The cvHaarDetectObjects function is used to search for objects using Haar cascades. The default value is 1.1. Preliminary experiments on used datasets quickly proved that the default value of this parameter gives the best results—a relatively fast detection time and a small number of false negative decisions. Decreasing the parameter value does not seem to make sense—it significantly increases the calculation time, while introducing many incorrectly detected faces. Increasing the parameter value shortens the detection time, however one should take into account the possibility of not detecting some objects. Acquiring data on objects appearing in the analysed frame is done by using the detectMultiScale method with the following parameters:

- image—input image for analysis, in a grey scale (8 bits per pixel),
- scaleFactor—mask scaling factor used to search for features in subsequent iterations of the algorithm,
- minNeighbors—the coefficient determining the search accuracy, takes integer values ≥ 1, the higher it is, the greater the accuracy,
- minSize, maxSize—parameters defining the minimum and maximum size of recognized objects, expressed in pixels,
- objects—an array of vectors describing the rectangular areas surrounding the found objects.

A similar analysis was made for the eye pair search stage. In this case, the most favourable pair of parameters turned out to be scaleFactor = 1.15 and minNeighbors = 3. As a result of using eye pair detection, the number of false detections has been reduced.

A key element of the recognition process is the proper cropping of the detected facial image based on the eyes_position and eyes_width parameters related to the position of the eyes and the percentage of face width in the cropped image. Based on the experiments performed on samples from the datasets, the following set of values of framing parameters checked in the study was determined: eyes_position = {0.25, 0.3, 0.35 and eyes_width = {0.75, 0.8, 0.95, 1}.

The final model extracts video frames with any given frequency, nevertheless conducted examinations showed that three frames per second is a satisfactory balance between system accuracy and performance.

To sum up, the face detection stage of the system is an important step for further face expression analysis. High detection accuracy was achieved by using additional verification based on eye detection, which reduces false acceptance. Moreover, the selection of effective parameters for the face recognition stage of the system is a task that requires testing the system’s operation on samples available in the three databases used as the quality and characteristics of the samples heavily affect each of the value presented above.

### Emotion classification model

The emotion classification model based on facial expressions was initially implemented using the GoogleLeNet network [38], which won the ImageNet competition in 2014, achieving efficiency that was slightly higher than the VGGNet network [39]. The GoogleLeNet architecture also has an advantage over the VGGNet model due to its size—the VGG network is over half a gigabyte in size, and the proposed architecture is less than 30 MB [38].

However, an attempt to replicate experiments on the ImageNet database using both networks revealed that the VGG network achieves better results in practice. These results meet the published results of experiments made in other implementations [3,40]. While it is not entirely clear where this performance difference stems from, we note the lack of critical details about training techniques and optimization available in the publication itself [38].

In practice, the VGG network family also shows another advantage over GoogleLeNet, in VGG having a much higher generalization efficiency. This can be seen in how VGG variants are often used in implementations that require transfer learning, such as fine-tuning or feature extraction. For these above reasons, the VGG 16 architecture was used as the basis of the emotion detection component of our proposed system.

Further modifications to the model based on results obtained during initial experiments with VGG 16 and its modifications using the ImageNet database include:

1. Doubling the number of filters learned by every convolutional layer, along with network deepening.
2. All convolutional layers in the network having a 3×3 filter.

The training data set we used consists of 75,807 samples, each of which has been scaled to an image in shades of grey sized 224 x 224. Facess were arranged spatially according to face features. For this purpose, the Simplex optimization algorithm was used with two translational parameters and one rotary alignment parameter. By using this procedure, faces have approximately the same size in each photo.

#### Data augmentation

Since the image samples are in raw, non-normalized RGB format, this means that the possible pixel values are in the range of 0–255, consequently data normalization and pixel intensity scaling were performed.

Despite the training size of our dataset, it is still insufficient to carry out deep network architecture design training. Therefore, both the offline data augmentation technique and the "on the fly" techniques were used. The number of training samples increases tenfold after using methods such as image rotation, image inversion and accidental disturbances of the Gaussian distribution. Each of the images in the training set was rotated by ±5° and ±10°, white Gaussian noise was added with variations of 0.001, 0.01 and 0.02.

#### Architecture

While the sigmoidal non-linear activation function is a highly popular choice in modern neural network, in recent years researchers have found that other activations, especially rectified linear activation, commonly referred to as ReLU, work better than the sigmoid unit.

This empirical observation may be due to the problem of the disappearing gradient in deep networks. For the sigmoidal function, the slope is zero for almost all input values. As a result, the gradient tends to zero for deeper networks. For the ReLU function, the slope is non-zero for a much larger part of the input space, which allows for propagation of non-zero gradients. A variant of the ReLU function also used in this work is Parametric ReLU (PReLU):

$$\begin{array}{c} f(x)=\{\begin{array}{cc} x & \mathrm{if}\;x>0,\\ 0.01x & \mathrm{otherwise} \end{array}\\ \mathrm{where}\\ \mathrm{for}\;a\leq 1\;f(x)=\max(x,ax) \end{array}$$ (1)

For the purpose of network training a variant of the RMSProp algorithm, the Adam algorithm, was used in this study. The Adam algorithm determines the adaptive learning coefficient via calculating exponentially weighted averages of both the gradient as well as its square for each parameter [41].

#### Implementation details

The chart below (Table 1) presents the network structure based on the modified VGG architecture:

**Table 1:** Modified VGG network used for emotion recognition.

Layer/Block	Type of Layer	Shape	Filter
input_1	Input Layer	224 x 224	None
block1_conv1	Conv2D	224 x 224	3 x 3
block1_conv2	Conv2D	224 x 224	3 x 3
block1_pool	MaxPooling2D	112 x 112	2 x 2
block2_conv1	Conv2D	112 x 112	3 x 3
block2_conv2	Conv2D	112 x 112	3 x 3
block2_pool	MaxPooling2D	56 x 56	2 x 2
block3_conv1	Conv2D	56 x 56	3 x 3
block3_conv2	Conv2D	56 x 56	3 x 3
block3_pool	MaxPooling2D	28 x 28	2 x 2
block4_conv1	Conv2D	28 x 28	3 x 3
block4_conv2	Conv2D	28 x 28	3 x 3
block4_poll	MaxPooling2D	14 x 14	2 x 2
Block5_FC	Fully Connected	56	None
Block5_FC	Fully Connected	7	None
block5_softmax	Softmax	7	None

After each CONV2D convolution layer there is activation and then batch normalization. The following components were used to build the VGG network:

**Table:** 

**Listing 1** Initialising the model	
start model	
1: enter input	
2: kernel_size ←3	//3x3 kernel
3: stride ←2	//2x2 stride
4: activation ←’PRELU’	//Parametric Exponential Linear Unit (PRELU)
5: padding ← ’same’	//input has same size as output
6: pool_size ←2	//2x2 pooling kernel
7: dropout_rate ←0.3	//drop 30% of nodes

Then, after the structure of the neural network is defined. The model is initialized with the input shape, so the input shape and the channel axis can be updated.

All four blocks of convolution layers were designed using the same pattern and can be represented as follows:

**Table:** 

**Listing 2** Four main blocks of the modified VGG network	
//Block 1: first CONV = > PRELU = > CONV = > PRELU = > POOL	
filters_0 ←matrix size 32	
input ←padding(input)	//pad
conv_0 ←input * filters_0	//do convolution
conv_0←activation(conv_0)	//apply activation function
conv_0←normalization(conv_0)	//apply batch normalization
filters_1 ←matrix size 32	
conv_1 ←padding(conv_0)	//pad
conv_1 ←conv_1 * filters_1	//do convolution
conv_1←activation(conv_1)	//apply activation function
conv_1←normalization(conv_1)	//apply batch normalization
conv_1 ←Max_pooling(conv_1)	//apply max pooling
conv_1 ←Dropout(conv_1)	//apply dropout
//Block 2: second CONV = > PRELU = > CONV = > PRELU = > POOL	
filters_2 ←matrix size 64	
conv_2 ←padding(conv_1)	//pad
conv_2 ←conv_2 * filters_2	//do convolution
conv_2←activation(conv_2)	//apply activation function
conv_2←normalization(conv_2)	//apply batch normalization
filters_3 ←matrix size 64	
conv_3←padding(conv_2)	//pad
conv_3 ←conv_3 * filters_3	//do convolution
conv_3←activation(conv_3)	//apply activation function
conv_3←normalization(conv_3)	//apply batch normalization
conv_3 ←Max_pooling(conv_3)	//apply max pooling
conv_3 ←Dropout(conv_3)	//apply dropout
//Block 3: third CONV = > PRELU = > CONV = > PRELU = > POOL	
filters_4 ←matrix size 128	
conv_4 ←padding(conv_3)	//pad
conv_4 ←conv_4 * filters_4	//do convolution
conv_4←activation(conv_4)	//apply activation function
conv_4←normalization(conv_4)	//apply batch normalization
filters_5 ←matrix size 128	
conv_5←padding(conv_4)	//pad
conv_5 ←conv_5 * filters_5	//do convolution
conv_5←activation(conv_5)	//apply activation function
conv_5←normalization(conv_5)	//apply batch normalization
conv_5 ←Max_pooling(conv_5)	//apply max pooling
conv_5 ←Dropout(conv_5)	//apply dropout
//Block 4: first set of FC	
filters_6 ←matrix size 64	
activation ←None	
bias ←0	
conv_6 ← Flatten (conv_5)	//Flatten
conv_6 = activation (conv_6 · filters_6 + bias)	//Convert to dense
activation ←’PRELU’	
dropout_rate ←0.5	//drop 50% of nodes
conv_6←activation(conv_6)	//apply activation function
conv_6←normalization(conv_6)	//apply batch normalization
conv_6 ←Dropout(conv_6)	//apply dropout

The first convolutional layer learns 32 filters sized 3 x 3. Then, PReLU is activated and batch normalization takes place. Next a convolutional layer replicates the strategy and learns an identical number of filters sized 3 x 3, followed by the next activation of PreLU types and batch normalization. Subsequently, maximum pooling and a dropout layers are applied with a probability of 20%. From block to block, the number of filters increases, so when the process goes deeper within the network structure, more filters are learnt.

The last layers are fully connected layers. Neurons in fully connected layers are fully combined with all activation functions in the previous layer, which can be seen in regular neural networks. Their activation functions can therefore be calculated by multiplying the matrix followed by bias offset. It is worth seeing that the only difference between fully connected and convolutional layers is that neurons in the convolutional layers are connected only to the local region at the input and that many neurons in the convolutional layers have common parameters. However, neurons in both types of layers still calculate dot products, so their functional form is identical. The fully connected input layer (Flatten)—takes the output of previous layers, "flattens" them and turns them into a vector, which can be the input point for the next stage.

The first fully connected layer learns fifty-six nodes. Subsequently, the batch normalization as well as PReLU activation are implemented. This part of the network can be represented as follows:

**Table:** 

**Listing 3** Block 5 of the modified VGG network	
//Block 5: second set of FC	
filters_7 ←matrix size 56	
activation ←None	
bias ←0	
conv_7 = activation (conv_6 · filters_7 + bias)	//Convert to dense
activation ←’PRELU’	
dropout_rate ←0.3	//drop 30% of nodes
conv_7←activation(conv_7)	//apply activation function
conv_7←normalization(conv_7)	//apply batch normalization
conv_7 ←Dropout(conv_7)	//apply dropout

The final step in this architecture is the implementation of a fully connected layer with a softmax layer to achieve seven probability classes for emotions.

**Table:** 

**Listing 4** Block 6 of the modified VGG network	
//Block 6: softmax classifier	
input classes	//number of output classes matrix
activation ←None	
bias ←0	
output ← activation (conv_7 · classes + bias)	//Convert to dense
activation ←softmax	//Softmax activation function for output
output ← activation(output)	
return model	

#### Tests of architecture variants

Obtaining a high classification neural network is often an iterative process. As such, a series of experiments in various configurations were made before settling on our final architecture for the 7-dimensional emotion detection model. Throughout this phase of our work we employed a cross-validation method to examine our models’ accuracy. The total set of samples was randomly separated into the training (90%) and testing (10%) sets. This process was iterated ten times and is presented below as an average percentage of identification accuracy, known in the literature as APIA [42]. This process tries to ensure that the model is generalizable, rather than simply fitting the data provided.

The first series of tests were conducted on an unmodified VGG network. The default Keras implementation settings of the VGG network were used for weight initialization in the convolutional as well as fully connected layers [43]. The SGD optimizer with a basic learning factor 1e - 3, Nesterov acceleration factor and momentum of 0.8 were used. Normalization and pixel intensity scaling was turned on, however no data augmentation was used in this iteration. After 35 epochs, the accuracy was 64.37%, and further training did not increase the model’s performance.

The second series of tests were conducted based on work presented in [41] with the optimization function changed to Adam. The Nesterov acceleration factor and the momentum of 0.8 were again used, as well as two learning factors 1e - 3 and 1e - 4. With these new settings accuracy increased by 7.21%. However, tests with both learning factors cause overfitting, with an increase in the validation loss rate, and the training losses decreasing simultaneously after every epoch greater than 60. This result indicates that there was a problem with the sample size. Sample size was increased by adding Gaussian noise with variations of 0.001, 0.01 and 0.02 as well as image inversion. These procedures increased the network accuracy level by 2.1% and partially solved the overfitting problem.

The third series of experiments tested various activation and initialization functions. All experiments were executed on fully augmented data, meaning that we performed additional rotation of the images in the training by ±5° and ±10°. The final sample number proved to be resistant to overfitting. The training procedure was repeated using the combinations of ELU, ReLU and PReLU functions together with the initializations of MSRA and Glorot. The highest performance and the shortest error reduction time were achieved with the combination of PReLu activation and MSRA initialization on the dataset that was fully augmented attaining 76.8% accuracy after 70 epochs.

During a thorough examination of the learning process for the final architecture, it is important to note that the PReLu function adapts more effectively to other parameters (such as weights and bias) by learning from lower values. This activation function learns the parameters of linear functions and improves precision, while resulting in a small additional calculation cost. Unlike traditional sigmoidal units, PReLU is not a symmetrical function. As a result, the PreLU response is never less than zero. Also, when assuming that inputs/weights are subject to symmetrical distribution, the response distributions can still be asymmetrical due to the behaviour of PReLU. These PReLU properties increase the consistency and performance of training compared to all other activation methods investigated.

The main difference between the initialization of MSRA and Glorot is that the first focuses on the non-linear nature of the function. Both methods are able to converge the VGG network, but MSRA begins to reduce the error earlier.

The maximum accuracy of the presented model is a 5% improvement when compared to the most widely used reference, which is the performance ranking attained in *Challenges in Representation Learning*: *Facial Expression Recognition Challenge* [44]. Table 2 presents a summary of previous research on video-based emotion recognition using IEMOCAP database.

**Table 2:** Summary of previous research on video-based emotion recognition using IEMOCAP database.

Ref	Method	Accuracy
[45]	Deep Belief Networks (DBN)	73.78%
[46]	ARTMAP Classifier	72.20%
[47]	Stacked CNN for Mocap Model combined with Bidirectional Long short-term memory (LSTM) with attention	71.04%
[48]	Emotion profiled Support Vector Machine (SVM)	71.00%
[49]	Informed Segmentation and Labelling Approach (ISLA) with addition of audio information	67.22%
[50]	SVM with Reynolds Boltzman Machine	60.71%
[51]	SVM speaker-inclusive (Sp-In) video (V) data	60.30%

### Determining conflict from emotion classifications

With a trained emotion classification model each input video frame is able to be classified across the 7 dimensions of emotion as defined above. Owing to our hierarchical two-stage classification approach, this vector of emotions is then used as the basis for prediction of the level of conflict.

Training of this conflict prediction model was completed using the SSPNet Conflict Corpus. SSPNet is a publicly available dataset of 1430 sample videos based on more than twelve hours of political debates. The video material presents reactions and behaviour of 138 debate participants. The data was extracted from forty five recordings of political debates televised by Canal 9 in Switzerland during 2005 [52,53]. The debates were divided into even segments of thirty seconds. It is worth mentioning that this data was also used as a benchmark for the "2013 Interspeech Computational Paralinguistic Challenge” [54].

This type of content can be considered as a valuable substitute to field data and as such is frequently utilized for the study of nonverbal behavioural patterns like facial expressions and more broadly physical expressions of emotions [55]. Given the context, political debate, the chances of detecting periods of conflict in investigated video materials are satisfactorily high.

Video was fed through the trained emotion classification model to firstly convert this video data into a time series of emotion classifications, after which correlations between these computer-generated emotion probabilities and the human-reported conflict levels were sought using a modified regression method. The regression procedure was executed multiple times to fine tune each step and achieve highest accuracy.

The implementation of the algorithms was performed in a Python environment using the Scikit-Learn, TensorFlow and Keras libraries. Additional data transformations, calculations as well as visual data analysis have been performed using SPSS [56].

### Data operations using a contrastive algorithmic approach

Given the size of the conflict training data, it is infeasible to additionally manually label each individual frame for emotion content. Instead the emotion classifications obtained through the level one classifier are used to provide labels for the conflict dataset. Given the presence of error in these emotion classifications (the emotion classifier is ~76.8% accurate), we refer to these emotion labels as ‘soft labels’. In addition, we analysed a 10% subset of the conflict dataset using a random selection strategy, to provide additional manual verification the presence of specific emotions, and used these results in our contrastive approach detailed below.

For the training of the level two classifier (conflict classification) we are thereby forced to find a solution which considers the presence of error in the soft labels of our input dataset. A promising solution is the innovative Contrastive Pessimistic Likelihood Estimation (CPLE) which was proposed in 2015 [57]. Please note that the notion “contrastive” is very often used in the area of machine learning and it usually specifies the condition which is achieved as a difference between two opposing restrictions.

Before proceeding to our specific implementation of the CPLE for measuring the degree of conflict in the analysed video samples, it is worth introducing the basic mathematical assumptions. Having a labelled dataset *(x*, *y)* consisting in the definite number *n* of the examples, we can define the function of the cost of the general estimator using the log of likelihood.

$$L(\bar{\theta };\bar{x},\bar{y})=\sum_{i}\log p(x_{i},y_{i}|\bar{\theta })$$ (2)

where $p(x_{i},y_{i}|\bar{\theta })$ is the likelihood that the label for the given example x_i_ is obtained after training the selected machine learning model. On the basis of the formula (2) it can be seen that the CPLE method constitutes a general structure which can be used in any classification algorithms if no probabilities are known. It has been stated on the basis of [58,59] that what is called Platt scaling [60] can be an efficient supplement to the earlier semi-supervised classifications, which allows for converting a decision function into probability using a parameterised sigmoid function. In the case of the classification this technique takes the following simplified form:

$$p(y_{i}=+1|x_{i},\bar{\theta })=\frac{1}{1+e^{\alpha f(x;\bar{\delta })+\beta }}$$ (3)

where the maximum likelihood method has been applied to the same training dataset, for the original classifier *f*, and parameters α and β have been learned for calculating maximum likelihood.

The CPLE algorithm outlines a contrastive condition by determining facilitation of the overall cost function in the semi-supervised model in comparison with the supervised solution using the following method:

$$L(\bar{\theta };X_{t},Q)-L(\bar{\theta_{s}};X_{t},Q)=CL(\bar{\theta },\bar{\theta_{s}},X_{t},Q)$$ (4)

Where *θ*_*s*_ denotes a learned supervised parameter. The term responsible for the supervised elements $L(\bar{\theta_{s}};X_{t},Q)$ has been developed further in the implementation in order to describe the elements with soft labels:

$$L(\bar{\theta };\bar{x},\bar{y})+\sum_{i=N+1}^{N+M}\sum_{k}q_{i}^{\left(k\right)}\log p(\bar{x_{i}^{u}},y_{i}^{u}=k|\bar{\theta })$$ (5)

We should remember, however, that these elements have been selected randomly at the beginning rather than being based on empirical observations, meaning the larger proportion of the labels (90%) have not been verified manually. For that reason, the ultimate goal in implementation is expressed in the following way:

$${\bar{\theta }}_{ss}={max}_{\bar{\theta }}CPL(\bar{\theta },{\bar{\theta }}_{s},X_{t},Q)$$ (6)

Where *θ*_ss_ indicates a semi-supervised parameter. The aim of the implementation on the dataset used here, which has been obtained using the level one classifier, is finding the best set of parameters, ensuring maximum accuracy, from the basic supervised threshold (calculated using supervised examples) to begin with, to its correction, taking into account the structural traits ensured by the marked examples. For that reason, another logarithmic likelihood function has been used as a pessimistic condition:

$$CPL(\bar{\theta },{\bar{\theta }}_{s},X_{t},Q)=\min_{x}CL(\bar{\theta },{\bar{\theta }}_{s},X_{t},Q)$$ (7)

In such an implementation the predict_proba method, restores the probabilities of belonging to a given class. Platt scaling is conducted automatically or on demand, depending on the classification of the method used.

### Implementation of regression in ill-conditioned experimental data

A crucial problem of the exploratory data analysis (EDA) in the case of the data obtained from the level one classifier is solving the problem of ill-conditioned data. When we are using polynomial logistic regression, entering a minor error, even in the case of one observation, affects the predicative capabilities of the trained model significantly. In the case of input, which have been generated by the VGG-based convolutional network model, the assumption that some variables are ill-conditioned is justified.

In the analysed multidimensional case *(p > 2)* it is hard to determine some incorrect points, which may be of substantial importance to an ill-conditioned system. Of course, there are adequate statistics on the basis of which it is possible to estimate this, also in the procedures of the multidimensional regression analyses [61,62]. In the below experiment a relatively simple method consisting in an adequate reduction of the number of independent variables occurring in the model has been applied. Let us refer to the polynomial model at this point. By replacing the data of any observed matrix with a linear equation, the sequence *n* of algebraic equations is obtained in the form of:

$$\begin{array}{c} y_{1}=b_{0}+b_{1}x_{11}+b_{2}x_{21}+\cdots +b_{p}x_{p1}+\varepsilon_{1}\\ \ldots \ldots \ldots \ldots \ldots \ldots \ldots \ldots \ldots \ldots \ldots \ldots \ldots \ldots \ldots \ldots .\\ y_{i}=b_{0}+b_{1}x_{1i}+b_{2}x_{2i}+\cdots +b_{p}x_{pi}+\varepsilon_{i}\\ \ldots \ldots \ldots \ldots \ldots \ldots \ldots \ldots \ldots \ldots \ldots \ldots \ldots \ldots \ldots \ldots .\\ y_{n}=b_{0}+b_{1}x_{1n}+b_{2}x_{2n}+\cdots +b_{p}x_{pn}+\varepsilon_{n} \end{array}$$ (8)

where ε_i_ are certain unknown errors, with which the above equations are satisfied via the values of the observed matrix.

By subtracting *y*_*i*_*−y*_*1*_ in turn for *i = 2*, *3*,…, *n*, a system of the equations *n -1* is obtained in the form of:

$$\Delta y_{i}=b_{1}\Delta x_{1i}+b_{2}\Delta x_{2i}+\cdots +b_{p}\Delta x_{pi}+\Delta \varepsilon_{i}$$ (9)

where Δ stands for a difference in the right variables. Then, dividing the Eq (9) by the variable Δ_x1i_ in turn for every i = 2, 3, 4,…, n, we obtain a system of the equations *n -1* in the form of:

$$\frac{\Delta y_{i}}{\Delta x_{1i}}=b_{1}+b_{2}\frac{\Delta x_{2i}}{\Delta x_{1i}}+b_{3}\frac{\Delta x_{3i}}{\Delta x_{1i}}+\cdots +b_{p}\frac{\Delta x_{pi}}{\Delta x_{1i}}+\frac{\Delta \varepsilon_{i}}{\Delta x_{1i}}$$ (10)

The system of the Eq (14) can be written in the form of:

$$Y_{i}=b_{1}+b_{2}X_{2i}+\cdots +b_{p}\delta_{pi}+\delta_{i}$$ (11)

where new variables *Y*_*i*_,*X*_2*i*_,*Z*_3*i*_,…,*X*_*pi*_ are defined as:

$$Y_{i}=\frac{\Delta y_{i}}{\Delta x_{1i}},\;X_{vi}=\frac{\Delta x_{vi}}{\Delta x_{1i}},\;\delta_{i}=\frac{\Delta \varepsilon_{i}}{\Delta x_{1i}}$$ (12)

where *ν = 2*, *3*,…, *p*. The new variables *Y*, *X* are substitution variables. As can be seen, the number of independent variables is reduced from *p* to *p -1*. The initial system *n* of the equations can be replaced with the system *n -1* of the equations in the form of (9), where the number of unknowns and independent variables is smaller by one.

This procedure has been repeated successively until such a number of the independent variables in an equation of regression has been obtained, which results in the smallest intended error of prediction model. In the case of the analysed video samples, the most effective solution, from the perspective of efficiency of the prediction model, was the reduction of the number of variables from the initial number p = 7 to p = 4.

#### Polynomial classifier

Given that the ill-conditioned data approach outlined above has not been described in the literature in detail, the choice of second-stage classification method has begun with a standard exploratory data analysis (EDA) technique. Among several classes of mathematical models of machine learning used in the EDA, the polynomials are of particular importance, and because the dependent variable in the analysed model is not limited to two categories it is used in the presented study. It is the result of their numerous advantages [63], and it seems worthwhile reminding the most important of them below:

- polynomials are smooth functions,
- a set of the polynomials $P_{m}$ of a degree not greater than *m* creates a linear space,
- polynomials are very well suited for numerical calculations (computing),
- the derivatives of polynomials are also polynomials,
- each continuous function defined in the range *[c*, *d]* can be, with any accuracy in the respect of a norm |⋅|_∞_, approximated with a polynomial (Weierstrass theorem),
- experiment matrices of the polynomial models are full rank (for various measurement points)

These advantages result in the fact that polynomials are the class tested if there are no preconditions allowing for a selection of the machine learning model. An important application of polynomials is an optimisation consisting in determining the minimising points via a cost function. Often the optimised object model is not known. Then, one of the basic optimisation methods is iterative method using polynomials. In the environment of the points given, a local polynomial model of an object is constructed and its optimal point is outlined. Then, in the environment of this solution, a new local model is constructed and the optimum is determined. This process is continued until a satisfactory solution is achieved.

Before proceeding to a further description of the method used, it is worth presenting the qualities of a polynomial model in a brief form. By applying $x_{ij}=x_{i}^{j}$ in the general linear regression model *y*_*i*_ = *b*_0_+*b*_1_*x*_*i*1_+⋯+*b*_*p*_*x*_*i*,*p*_+ε_*i*_, we obtain a polynomial model:

$$\begin{array}{c} y_{i}=b_{0}+b_{1}x_{i}+\cdots +b_{p}x_{i}^{p}+\varepsilon_{i}\\ \mathrm{and}\\ i=1,\ldots ,n \end{array}$$ (13)

In this way the experiment matrix has the form of:

$$X=\left[\begin{array}{ccccc} 1 & x_{1} & x_{1}^{2} & \ldots & x_{1}^{p}\\ 1 & x_{2} & x_{2}^{2} & \ldots & x_{2}^{p}\\ \ldots & \ldots & \ldots & \ldots & \ldots \\ 1 & x_{n} & x_{n}^{2} & \ldots & x_{n}^{p} \end{array}\right]$$ (14)

hence, the (*rs*)-th element of the matrix *X*^*T*^ *X* equals ${\left(X^{T}X\right)}_{rs}=\sum_{i=1}^{n}x_{i}^{r+s}$. If elements *x*_*i*_ are equally spaced over the range ⟨0,1⟩, then for determining the weight of the big *n*, the (rs)-th element of the matrix can be written as [64]:

$$\begin{array}{l} {\left(X^{T}X\right)}_{rs}=n\sum_{i=1}^{n}x_{i}^{r}x_{i}^{s}\frac{1}{n}\\ \approx n\int_{0}^{1}x^{r+s}dx \end{array}$$ (15)

where *s* is the sample standard deviation and *r* is the differentiating vector [*r*_1_,…,*r*_*n*_]^*T*^ of observations *y*.

It follows that the matrix *X*^*T*^ *X* is by approximation equal to the matrix *H*_*p+1*_ whose dimension is *(p + 1) × (p + 1)* lying in the right left corner in the Hilbert matrix. The conditioning of the matrix ***A*** is a conditional number defined by the formula *κ*(***A***) = |***A***||***A***^−**1**^|. For a symmetrical matrix positive definite it amounts to a quotient of maximal λ_max_ to minimal λ_min_ of the value of the own matrix *A*, κ(*A*) = λ_max_/λ_min_. The conditional number of the unitary matrix is 1, and for the degenerate matrix ∞.

It can be easily seen that the conditional number of the matrix *X*^*T*^ *X* of a polynomial model increases quickly with the growth of the degree of a polynomial, e.g. for a polynomial with the degree of k = 10, the conditional number is 3 · 10^10^ [65], and it means that the errors resulting from the calculations increase 3 · 10^10^.

The numerical problems related to incorrect matrix conditioning increase quickly along with the growth of the degree of a polynomial. They can be of vital importance for a polynomial with the degree of five already. In order to prevent this, specific computational algorithms and/or orthogonal polynomials should be used. Several classes of algorithms as well as orthogonal polynomials have been drawn up, and they can be applied in various ways [65,66]. The implementation which has been used in the experiment described is implementation tf.nn.softmax [67]. In the case of loss calculation function the method of cross entropy has been used. Again, TensorFlow implementation has been used instead of the manual defining of cross entropy in order to avoid complex numerical problems developing while calculating loss.

#### Optimisation

Based on the experiments described in [41], the optimisation has been obtained via Adaptive Moment Estimation algorithm, which has been created in order to further improve the effectiveness of RMSProp algorithm. It determines the adaptive learning coefficient via calculating exponentially weighted averages of both the gradient as well as its square for each parameter.

$$\begin{array}{c} {\bar{g}}^{\left(t+1\right)}({\bar{\theta }}_{l})=\mu_{1}{\bar{g}}^{\left(t\right)}({\bar{\theta }}_{l})+(1-\mu_{1})\nabla_{\bar{\theta }}L({\bar{\theta }}_{l})\\ \mathrm{and}\\ {\bar{v}}^{\left(t+1\right)}({\bar{\theta }}_{l})=\mu_{2}{\bar{v}}^{\left(t\right)}({\bar{\theta }}_{l})+(1-\mu_{2}){\left(\nabla_{\bar{\theta }}L({\bar{\theta }}_{l})\right)}^{2} \end{array}$$ (16)

In the described experiment these parameters have the following values:

beta_1 = 0.9, beta_2 = 0.9, decay = 1e-2, epsilon = 1e-6, lr = 0.0001

The forgetting factors μ_1_ and μ_2_, are represented by parameters beta_1 and beta_2 in this case. In the case of the elements in decay, epsilon and lr standard values have been kept for other algorithms.

As it has been suggested by the authors, in [41] two calculations (regarding moments one and two) should be relieved by their division by the value 1 – μ_1_, and because of that the new moving averages are as follows:

$$\begin{array}{c} {\hat{g}}^{\left(t+1\right)}({\bar{\theta }}_{l})=\frac{{\bar{g}}^{\left(t+1\right)}({\bar{\theta }}_{l})}{1-\mu_{1}}\\ \mathrm{and}\\ {\hat{v}}^{\left(t+1\right)}({\bar{\theta }}_{l})=\frac{{\bar{v}}^{\left(t+1\right)}({\bar{\theta }}_{l})}{1-\mu_{1}} \end{array}$$ (17)

Where the weight update rule in the algorithm is:

$${\bar{\theta }}_{l}^{\left(t+1\right)}={\bar{\theta }}_{l}^{\left(t\right)}-\frac{\eta g^{\left(t+1\right)}({\bar{\theta }}_{l})}{\sqrt{v^{\left(t+1\right)}({\bar{\theta }}_{1})+\delta }}$$ (18)

Like all methods based on momentum, the algorithm under discussion can result in the instability (oscillation) while learning. However, in the case of the experiment in question it has demonstrated high effectiveness while remaining stable for the small values of the learning coefficient.

## Results

### Baseline classification accuracy

**Fig 2:**
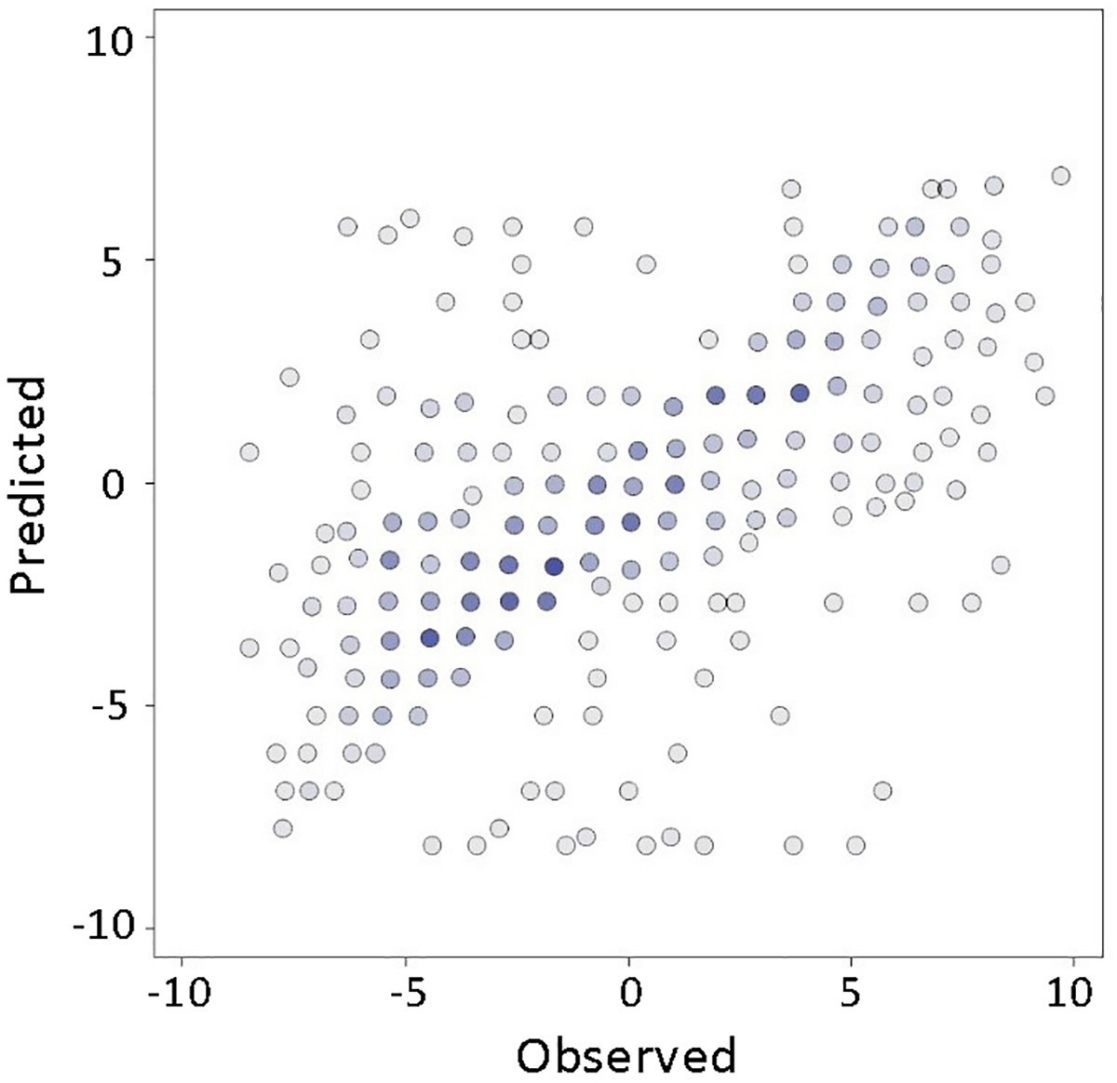
The accuracy of polynomial model without implementation of CPLE nor dimensionality reduction.

A standard tenfold cross-validation method was used to estimate our proposed conflict detection models’ accuracy levels. In the case of the first method, without implementation of CPLE and dimensionality reduction, the average score of the model was 52.3% which is not satisfying by any means for polynomials (Fig 2).

**Fig 3:**
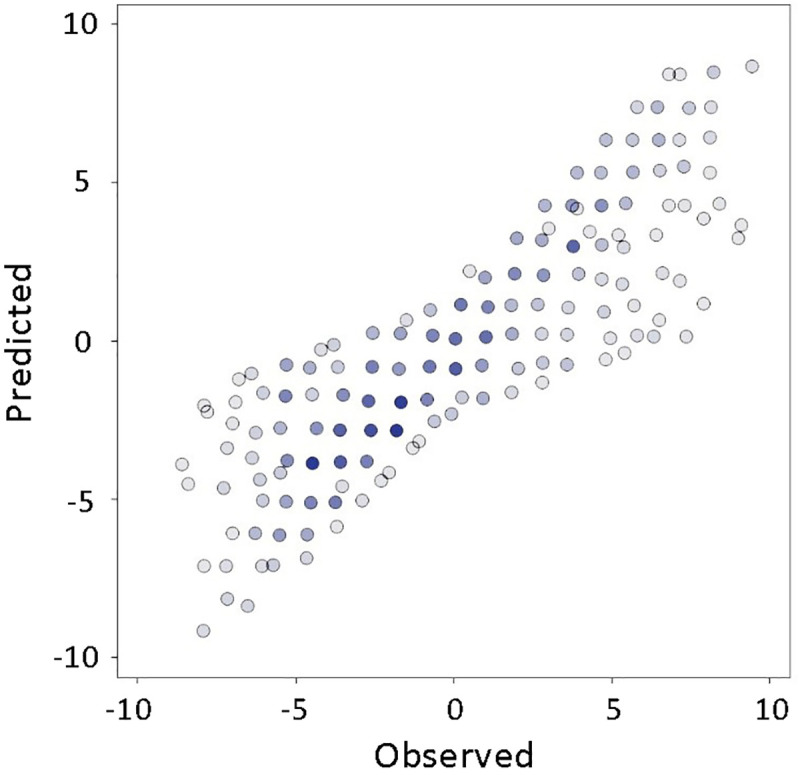
The accuracy of polynomial model with implementation of CPLE but without dimensionality reduction.

Our novel treatment of the second level classifier training as a semi-supervised problem through introduction of a CPLE algorithm to the data pre-processing and transformations resulted in an increased average prediction score to 77.4% (Fig 3).

**Fig 4:**
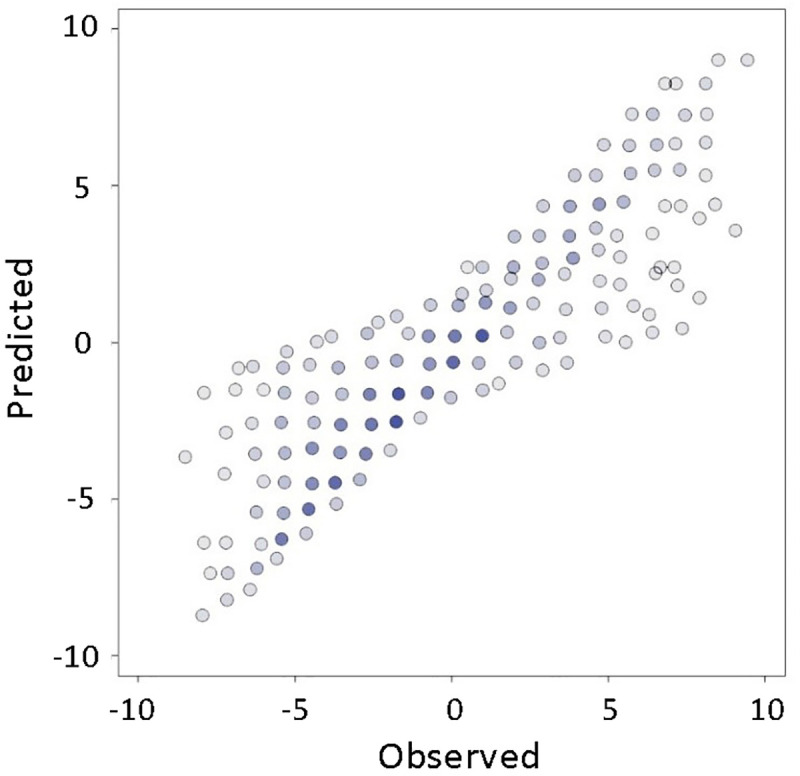
The accuracy of polynomial model with both CPLE algorithm and dimensionality reduction method implemented.

The final prediction accuracy score is achieved by combining a semi-supervised method with an additional assumption of so called ill-conditioned experimental data. The average score is 80.7% (Fig 4).

Before any modelling was conducted, the data was cleaned using missing values replacement and outlier identification. After removing all of identified influential outlying data, it was established that the parameter estimates are defined by the majority of the cases, instead of just by an infrequent or inordinately influential small data subsection. This step of data preparation for linear regression was essential as it is well known that a minor subgroup of the data can have a disproportionate impact on the estimated parameters and predictions, therefore there is a frequent concern that the model estimates are based primarily on small data subset rather than on the majority of the data to not jeopardize the accuracy of main analysis.

The study utilized a two-step process to detect model fit outliers. The first step involved detecting cases that are expected to have impact on the model fit as they diverged distinctly from other data points in the overall set. The basis for the first step was a practical one since this activity decreased the amount of data points to which the more laborious second step must be used. The second step involved inspecting previously detected data points to recognize if they have actual effect on model fit. This was to establish if data points that vary decidedly from the rest impact fit of the model (R2). This included examining if the exclusion of an observation affects the statistical significance of a fit of the model ranked from statistically non-significant to statistically significant [68].

### Broadcast interview data analysis

We selected three broadcast interviews, drawing mostly from examples of accountability interviews, two from Channel 4 (UK), and one BBC. These interviews were selected as they are prototypical of the kind of interviews that are of interest to broadcast discourse analysis scholars. The interviews are:

- Guru-Murthy/Tarantino: Krishnan Guru-Murthy interviews film director Quentin Tarantino on Channel 4 News. Movie promotional interview. Director declines to discuss the link between onscreen and real-life violence. Published on 10^th^ January 2013. Duration 8 minutes 35 seconds.
- Newman/Yiannopoulos: Cathy Newman interviews controversial alt-right figurehead Milo Yiannopoulos on Channel 4 News. Political interview. Milo Yiannopoulos gives his perspective on wage gap and gender inequality. Published on 18^th^ November 2016. Duration 5 minutes 43 seconds.
- Paxman/Howard: Jeremy Paxman interviews Michael Howard on BBC Newsnight. Political interview. Presenter challenges the former British Home Secretary over the divisive removal from office of the head of the Prison Service. The interview is broadly known as one of the most eminent confrontations between a journalist and politician in British television history. First broadcast on 13^th^ May 1997. Published on 8^th^ October 2015. Duration 8 minutes 10 seconds.

#### Determining a ground truth for conflict levels

After the creation and training of our automated classifier, but independent to the calculation of conflict measures in the broadcast interview case studies via our automated approach, three independent human experts performed a series of manual annotations on the three broadcast interviews. The broadcast interviews were divided into 15 second intervals. Then, each interval was assessed in terms of its perceived conflict level. In total, the experts each assessed 89 intervals of 15 seconds each. The aim of this analysis was to obtain proper reference to automatic annotation performed by the presented system, known as a ground truth. The “ground truth” term, in machine learning, refers to the accuracy of the set of examples for the supervised techniques of machine learning. Ground truth is indispensable in statistic models to prove or reject a research hypothesis. As a process, ground truth refers to the collection of proper data and verification of their accuracy in order to determine the degree of the classification efficiency of the statistic models obtained in machine learning.

To estimate the accuracy of the human-based coding, inter-coder reliability using Cronbach’s alpha was applied. A value of 0.893 was obtained which can be interpreted as a strong correlation, thus constituting a reliable ground truth for measures of speaker conflict and later comparison with our system derived values.

#### Case Study 1: Michael Howard and Jeremy Paxman, Newsnight

This first case study is used to explore the extent by which it is possible to pinpoint the initiation, amplification, moderation, and eventual conclusion of conflict in this interview through the lens of our computational classification approach?

**Fig 5:**
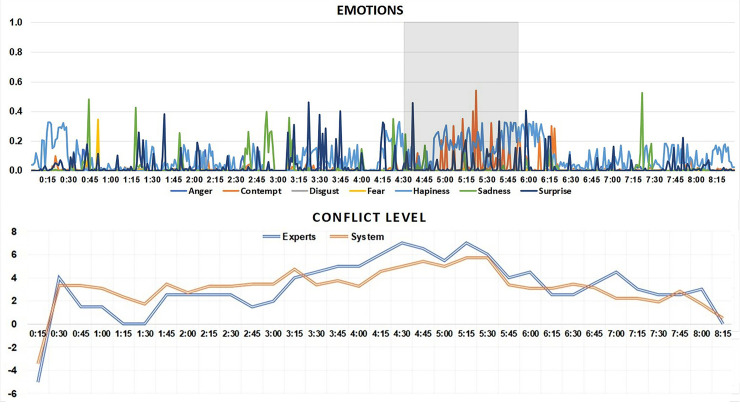
Results for Interview 1, Jeremy Paxman interviews Michael Howard on BBC Newsnight. This figure shows results from the automated system classification of emotion and conflict (axes Y). Axes X represent time points within the video.

This famous Newsnight interview makes an ideal first case study due to the manner in which Paxman’s pattern of near verbatim question repetition is understood to impact on the sense of tension and conflict inside the studio. Human ratings of the level of conflict in this case study reveal that a significant proportion of the conversation has a low to moderate level of conflict, but that there is a period of increased and sustained conflict from 3:00 to 6:15 (Fig 5). The results from the automated system classification of emotion and conflict are also produced in for comparison against these human ratings.

The baseline system predicted conflict level tends to situate around 3 (remembering it is bounded to a maximum conflict level of 20, and minimum of -20). The conflict level rises at around 3:00, corresponding to the following excerpt of the interview (2:50–3:40):

JP: Derek Lewis says “Howard had certainly told me that the Governor of Parkhurst should be suspended, and had threatened to overrule me”. Are you saying Mr Lewis is lying?
MH: I have given a full account of this, and the position is what I told the House of Commons, and let me tell you what the [position is]
JP: [So you are] saying Mr Lewis is [lying]
MH: [let (.) let] me tell you exactly what the position is. I was entitled to be consulted (.) and I was consulted, I was entitled to express an opinion, I did express an opinion, I was not entitled to instruct Derek Lewis what to do, and I did not instruct him what to [do =
JP: [well his ver-]
MH: = and] you will understand and recall that Mr Marriot was not suspended, he was moved:: and Derek Lewis told the Select Committee of the House of Commons, that it was his opinion, Derek Lewis’ opinion, that he should be moved immediately. That is what happened.

The section above is the first moment of the interview where Paxman begins his line of repeated questioning regarding whether Michael Howard “threatened to overrule” Derek Lewis. The now famous sequence of Paxman repeating his question continues until 5:45 at which point both Paxman and Howard can be seen with wry smiles at the almost comical nature of the repeated questioning. At 6:00 Paxman moves onto a new line of questioning regarding Howard’s leadership ambitions.

The episode of conflict in this interview can be bracketed in the conflict levels corresponding to 3:00–6:00. The key initiation point by Paxman: “Are you saying Mr Lewis is lying?” can be seen as a trigger for this sequence, while at 5:50 Paxman concludes this section of the interview by saying “right, we’ll leave that aspect there”. One can note the return of the conflict level back to its baseline of 3 at this point of the interview.

Interestingly, the emotions detected by the system that feed into the prediction of conflict here comprise of a triad of contempt, surprise and happiness. This is important to note as there is not a sense of hostility, or overt aggression in this interview, instead at times a sense of whimsy, and incredulousness at the insistence by Paxman with regard to his line of questioning, and of Howard’s refusal to answer.

#### Case study 2: Quentin Tarantino and Krishnan Guru-Murthy, Channel 4

This interview was selected due to the general heightened level of conflict, stemming from Guru-Murthy pursuing a line of questioning relating to Tarantino’s use of violence in his films, and Tarantino not wishing to be interviewed on this topic. It is an interesting interview from a broadcast talk genre perspective as well, given that Tarantino could be considered as fulfilling the requirements as an expert guest (as movie director), however much of Guru-Murthy’s questioning and interaction is of an accountability style.

**Fig 6:**
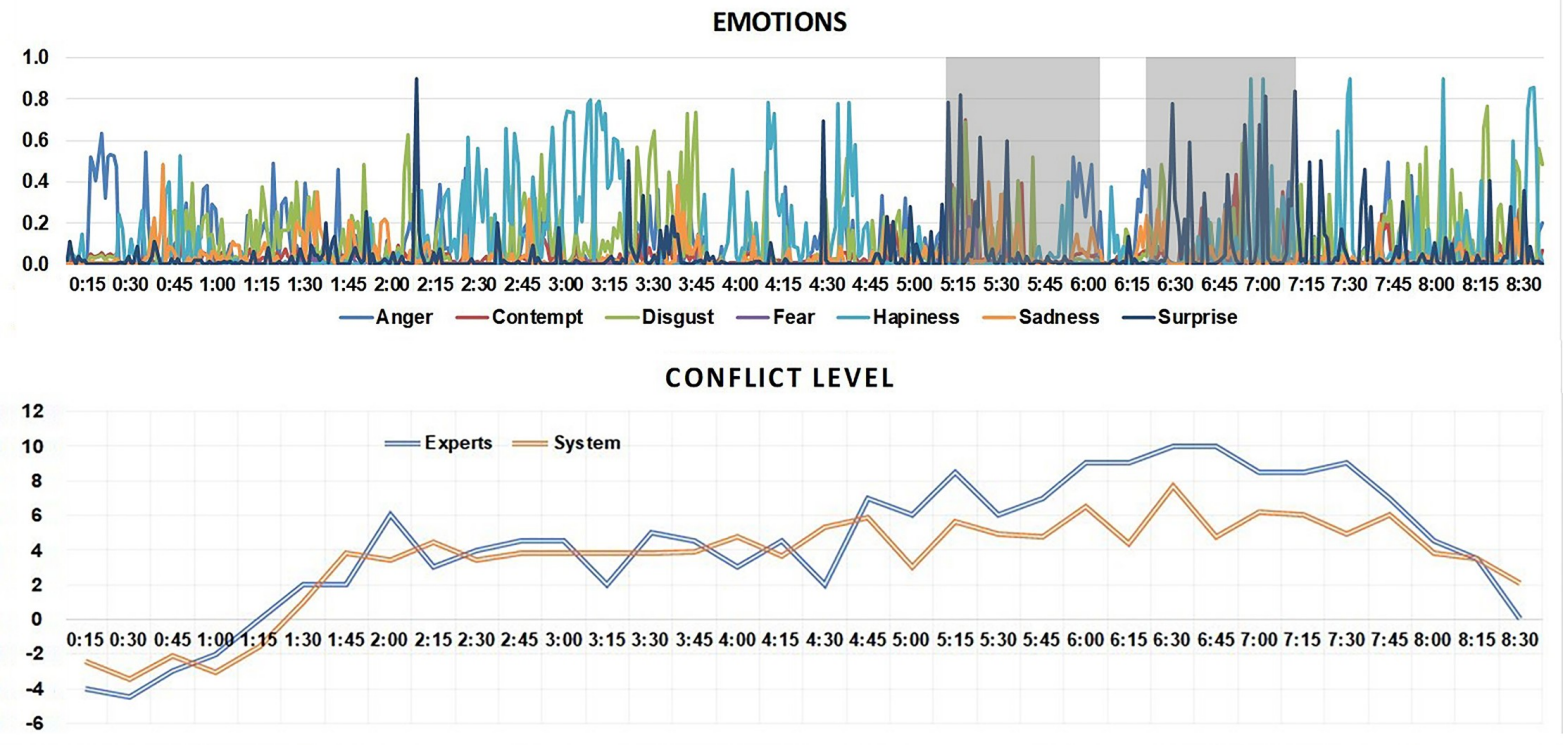
Results for Interview 2, Krishnan Guru-Murthy interviews film director Quentin Tarantino on Channel 4 News. The figure shows results from the automated system classification of emotion and conflict (axes Y). Axes X represent time points within the video.

Examining the emotion and conflict levels in Fig 6, we can observe that the first minute and a half is seen as having sub-zero conflict, indicating that this section of interview is more positive or neutral. This first section of the interview is largely Tarantino exploring his motivations for making his recent film with Guru-Murthy not challenging Tarantino’s claims and instead allowing him room to develop his own monologue. This positive/neutral stance lasts until time 1:15–1:30 where Guru-Murthy asks a question relating to the critical reception of Tarantino’s films and begins to open up the topic of his use of violence in his filmmaking.

The steady rise in conflict from this point seems to draw from a clash over the purpose of the interview itself, and Tarantino’s question refusals often engage in a meta-commentary of the discourse, such as with this excerpt from 4:30–4:48:

KGM: But why are you so sure that there is no link between enjoying movie violence, and enjoying real violence.
QT: I don’t. I. I’m going to tell you why I’m so sure? Don’t ask me a question like that, I’m not biting. I refuse your question.
KGM: Why?
QT: Because I refuse your question, I’m not your slave and you’re not my master. You can’t make me dance to your [tune =
KGM: [I can’t]
QT: = I’m not] a monkey.

Despite Tarantino’s refusal to address questions relating to violence and his movies, Guru-Murthy persists with this line of questioning, sustaining what are seen amongst the highest levels of conflict for the interview (5:45–5:57):

KGM: No but you y- you haven’t fleshed it out. [That was the re-]
QT: [I have- It’s not] my job to flesh it out.-
KGM: No, it’s my job to try to ask you to-
QT: And I’m shutting you butt down. [(laughter)]
KGM: [And that’s entirely your (.)] [your right]
QT: <[This is a] commercial for my movie>

The conflict peaks at 8 (human rating was 10) around 6:30 which corresponds to the following excerpt, still on the subject of Taratino’s views on violence in films:

QT: And you know where I stand on it
KGM: Which is that there’s no relation[ship]
QT: [Yes]
KGM: But you haven’t said why you think there’s no relationship, [(unclear)]
QT: It’s none of your damn business what I think about that (.)
KGM: Ur (.) Well it’s my job to ask you why you think [that because you’re very influential]
QT: [And I’m saying no] (2.0) And I’m shutting you down.

From this point we see a de-escalation of conflict with Guru-Murthy no longer probing into the topic of movie violence.

An examination of the emotions elicited by the emotion detection system, it appears that fear and surprise correlate, as do anger and disgust. These individual pairs correlate with heightened conflict levels, particularly when both pairs occur all at once. The expression of anger is perhaps easier for our system to predict given that it manifests in all three facial regions, namely the forehead, mouth, and eyes.

#### Case Study 3: Yiannopoulos and Newman

The final interview under consideration was selected because the interviewee, Milo Yiannopoulos, is a reactionary who often seeks to provoke outrage and antagonistic reactions from the media and audience to build his specific brand of infamy. While provoking his interviewers, Yiannopoulos himself tends to act various emotional roles, often feigning disgust, surprise, and anger. The interview analysed here was an accountability interview, with the interviewer Cathy Newman challenging many claims and performing a strong gatekeeping role throughout.

**Fig 7:**
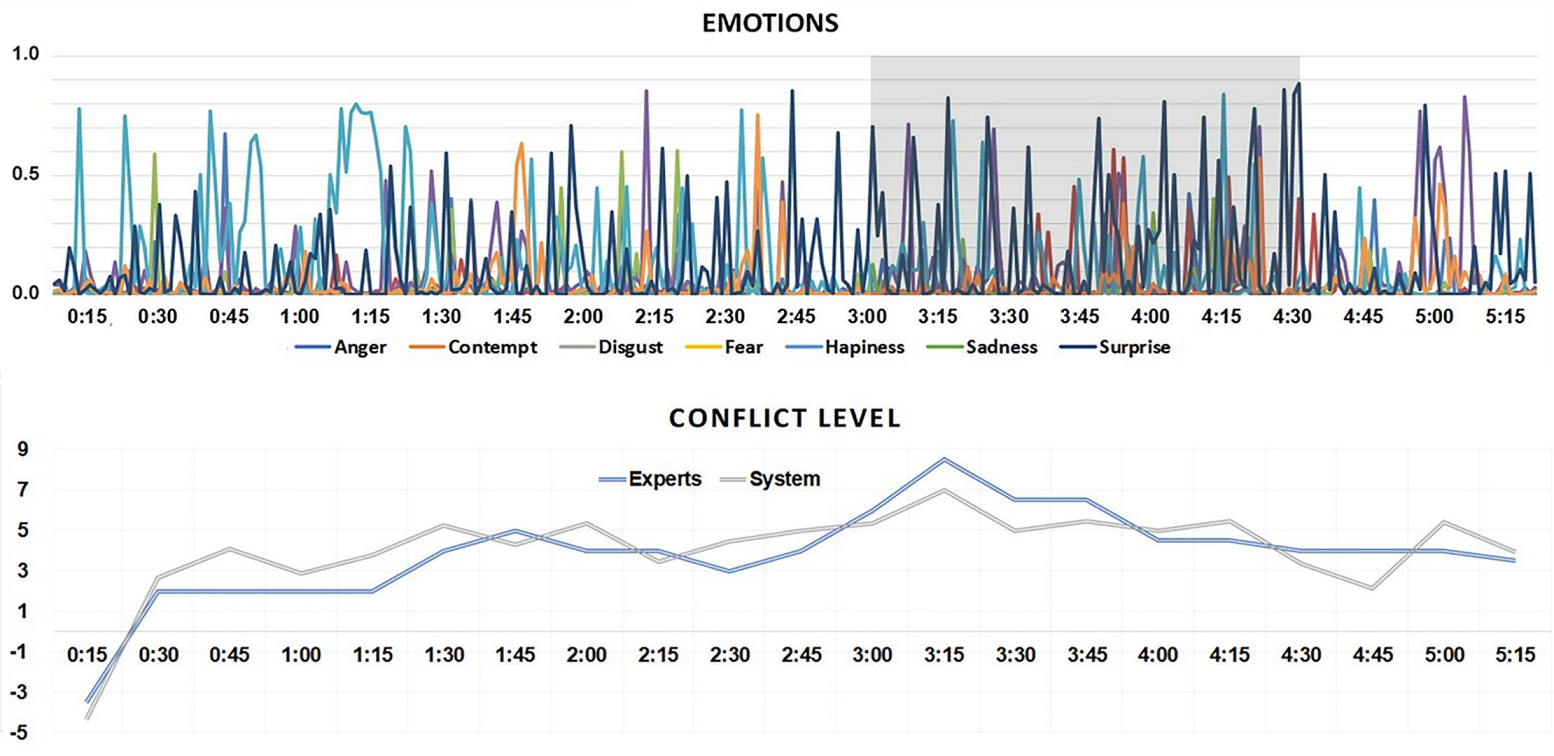
Results for Interview 3, Cathy Newman interviews controversial alt-right figurehead Milo Yiannopoulos on Channel 4 News. The figure shows results from the automated system classification of emotion and conflict (axes Y). Axes X represent time points within the video.

The results shown in Fig 7, reveal that contempt and disgust were the dominating pairs of emotions in this interview, and happiness blended with surprise was also observed. The interview is in many ways quite difficult to analyse given that much of the discourse features both interviewer and interviewee speaking over the top of each other. The peak period of conflict as detected by our system is one such example of this pattern of behaviour, featuring extensive overlap (3:00–3:16):

CN: No, I want to put some of that [satire to you =
MY: [Don’t you find (.)
CN: = I want to put some of your own satire to you =
MY: = Don’t you find it bizarre that Breitbart news.
CN: = Wait a minute, I know, I know =
MY: = Supposedly this. Supposedly this evil right-wing hate site, it publishes a gay dissident edu-]
CN: = I know you want women to log off the Internet but we are now in the Channel 4 news studio (.) you have to allow me to speak.]

## Discussion

In this paper we have outlined a machine learning based approach for computational classification of emotion, and a higher order emotional variant (conflict) within broadcast discourse. Our goals for this study were two-fold, firstly to propose a new qualitative discourse analysis support technology, and secondly to offer an introduction to deep neural network technologies which underpin our approach to the classification of emotion in broadcast discourse recordings.

The case studies showcased an overall high degree of agreement between our computational model and human expert ratings of conflict. The case studies also revealed how this new method could be used to help guide analysis and understanding regarding the onset, amplification, sustainment, and resolution of conflict in such discourse. In each case study the method was able to highlight moments of heightened conflict, and pinpoint their onset and conclusion.

To be fair, these moments of conflict are also readily identified by a human expert, and it was precisely for this reason that they were selected. However, in moving forward our method provides a model for how cross-validation of human codings could be performed in cases where extra human labour may be unavailable for the determination of inter-coder reliability. In other words, a human and this machine-based tool could each code a discourse, and the results compared in a similar way as we might compare two independent human codings.

Setting our sights further though, this method could be used to identify moments of conflict or other interesting emotional composites across vast volumes of recorded discourse, and used as a way to filter and highlight instances that could prove interesting for closer analysis and interpretation.

Owing to the second goal of this research, regarding the introduction and explanation of such advanced computational methods within the discourse analysis space. It is useful to consider how this machine-based approach carries with similar concerns to the manner in which we go about our work as human analysts of discourse. The definitions of the various emotional states used in this paper carry with them similar subjectivity and bias inherent in many other observable discourse phenomena that we may find interest in describing. The success of this computational classification method is tied to the quality of the input examples provided, in much the same way that our skills in describing a discourse phenomenon are tied to the robustness of our definitions, and provision of clear and diverse examples from real-world interactions containing these various behaviours.
